# A randomized trial evaluating the association between related gene polymorphism and nausea and vomiting induced by cisplatin multi-day chemotherapy

**DOI:** 10.1186/s12920-023-01719-0

**Published:** 2023-11-03

**Authors:** Yilan Jin, Feng Chen, Juan Zhao, Ying Jiang, Gaowa Jin, Zewei Zhang, Quanfu Li

**Affiliations:** 1 Department of Infectious diseases Department, Inner Mongolia People’s Hospital, Hohhot, 010017 China; 2 Department of Medical Oncology, Ordos Central Hospital, 23th Yijinhuoluo Western Road, Ordos, 017000 China; 3 Department of Radiation Oncology, Sun Yat-sen University Cancer Center, Dongfeng East Road, Guangzhou, 510060 China

**Keywords:** CINV, Gene Polymorphism, Cisplatin, Multi-day chemotherapy

## Abstract

**Purpose:**

We aim to investigate the correlation between gene polymorphisms and cisplatin chemotherapy-induced nausea and vomiting (CINV), which was prevented by olanzapine or aprepitant triple antiemetic regimen.

**Methods:**

Before chemotherapy, the blood samples of 89 malignant tumor patients who received multi-day chemotherapy with cisplatin were collected for sequencing and typing. As there were duplicate patients enrolled in different chemotherapy cycles, there were a total of 190 cases. The patients were divided into two groups randomly, who received the triple antiemetic regimen of olanzapine or aprepitant combined with 5-HT3RA and dexamethasone. The main evaluation indicators were the total protection (TP) rate in the acute phase (0–24 h), the delayed phase (25–120 h) and the overall phase (0-120 h).

**Results:**

Univariate analysis was performed on genetic loci that reached H-W balance with TP. In the olanzapine group, increased TP in the acute phase was associated with *HTR3A* rs1176719 non-GG (*P* < 0.05) genotype etc. Increased TP in the delayed phase was associated with *HTR3A* rs1176719 non-GG (*P* < 0.05) genotype etc. In the aprepitant group, increased TP in the acute phase was associated with the *MTHFR* rs1801131 TT (*P* < 0.05) genotype etc. Increased TP in the delayed phase was associated with *HTR3A* rs1062613 CC (*P* < 0.05) genetype ect. Multivariate Logistic regression analysis showed that *HTR3B* rs7943062GG (*P* < 0.05) genotype etc. were correlated with increased TP in the delayed phase. *MTHFR* rs1801131TT genotype was associated with increased TP in the acute phase (*P* < 0.05) and delayed phase (*P* < 0.05).

**Conclusion:**

This study found that gene polymorphisms, including *HTR3B* (rs1062613, rs1176719, rs2276303), *HTR3B* (rs45460698, rs7943062), *HTR3C* (rs6766410), *ERCC1* (rs3212986), *ERCC4* (rs744154) and *MTHFR*(rs1801131), may be independent prognostic factors for CINV.

**Supplementary Information:**

The online version contains supplementary material available at 10.1186/s12920-023-01719-0.

## Introduction

CINV can lead to electrolyte imbalance, dehydration, malnutrition, and esophageal damage, which not only affects the quality of life of patients, but also reduces overall survival and increases treatment costs [1]. A review of clinical trials identified several clinical risk factors for CINV: female sex, younger age, history of low-dose alcohol consumption and dizziness [2]. Individual differences in CINV occurrence, however, cannot fully and accurately be explained by these factors [3]. Single nucleotide polymorphisms (SNPs) are of great significance to the risk and individualized prediction of CINV. Different types of CINV (i.e., acute, delayed, predicted, sudden, and refractory) transmit via different pathways and neurotransmitters. Therefore, pharmacological approaches to prevention and treatment vary according to the type of CINV and the genes involved [4]. The gene profile among patients may be an independent risk factor. Related studies have shown that 5-hydroxytryptamine receptor 3(*5-HTR3*), excision repair cross-complementation (*ERCC*), methylenetetrahydrofolate reductase(*MTHFR*), ATP binding cassette subfamily G member 2 (*ABCG2*), Fas cell surface death receptor (*FAS*), C-C motif chemokine ligand 2(*CCL2*), ATPase copper transporting beta(*ATP7B*), Tachykinin receptor 1(*TACR1*), cytochrome P450 family2 subfamily D member 6(*CYP2D6*) and aldehyde dehydrogenase 2(*ALDH2*) gene may be associated with CINV.

Among the 5-HT and its receptor, only 5-HTR3 as a ligand-gated ion channel plays a role in the pathogenesis of CINV [5]. This receptor is involved in the transmission of information in the gastrointestinal tract, and regulates intestinal motility, inducing the occurrence of nausea and vomiting [6]. 5-HT3 receptor antagonists selectively bind to and inhibit 5-HT3R and are currently used to prevent and treat CINV after FDA approval, such as ondansetron and palonosetron. 5-HTR3 consists of subunits encoded by the *HTR3A*, *HTR3B*, *HTR3C*, *HTR3D*, and *HTR3E* genes [7]. Different subunit compositions lead to the complexity of the 5HT3 receptor system. Clinical and cell culture studies have found that variations in 5-HTR3 influence protein function and clinical outcome in CINV [8]. ERCC is an essential step in nucleotide excision repair pathway (NER). Cancer cells that express high levels of ERCC proteins and genes are more susceptible to chemotherapy toxicity and cisplatin resistance. ERCC1 and ERCC4 are key elements in the NER pathway [9]. It is currently believed that *ERCC1* polymorphisms may be associated with survival outcomes and gastrointestinal toxicity in patients receiving platinum-based chemotherapy. A key component of folate metabolism, *MTHFR* oversees gene regulation and DNA methylation [10]. A Chinese study found that *MTHFR* gene polymorphisms are associated with CINV [11]. The polymorphisms of drug transporter *ABCG2* and *ATP7B* genes may change the uptake and efflux rate of chemotherapeutic drugs into the blood-brain barrier, resulting in different incidence and severity of CINV [12, 13]. The transmembrane protein encoded by *ABCG2* gene is a part of the blood-brain barrier, which can lead to the outflow of some chemotherapeutic drugs [13]. *ATP7B* gene encodes ATP7B enzyme. The high expression of *ATP7B* gene is related to the higher outflow and accumulation rate of chemotherapeutic drugs in the blood. *FAS* and *CCL2* genes play an important role in controlling cell homeostasis. *CCL2* is a chemokine gene involved in immune regulation and inflammatory processes [14]. *FAS* is a death receptor system gene, which can mediate apoptosis induction to maintain immune homeostasis [15]. They are also important in the immune response and elimination of abnormal cells and cancer cells. Neurokinin 1 antagonists such as aprepitant exert antiemetic effects in the area postrema and nucleus tractus solitarius. A Japanese study suggested that the *TACR1* gene encoding the NK1 receptor may be related to CINV. ALDH2 is the key rate-limiting enzyme for the oxidative detoxification of acetaldehyde, the metabolite of ethanol [16]. A new Chinese study suggests that the rs671 mutation of the *ALDH2* gene may be a relevant factor affecting the occurrence of CINV [17].

This study is based on our previous study of *ABCB1* rs1045642, female is an independent risk factor for CINV [1]. We combined the 42 SNPs of metabolic enzymes, transporters and targeting receptors reported in domestic and foreign literatures that may be related to CINV to explore the relationship between related SNPs and CINV susceptibility. This provides a scientific basis for exploring cost-effective and individualized antiemetic solutions.

## Materials and methods

### General information

A group of patients who visited Ordos Central Hospital’s Department of Medical Oncology between March 2019 and December 2020 was collected. They were administered the highly emetogenic chemotherapy drug cisplatin in divided doses. Before chemotherapy, 10ml of peripheral blood was drawn from 89 patients, whose treatment consisted of olanzapine or aprepitant coupled with dexamethasone and 5-HT3RA randomly selected by random number table. As there were duplicate patients enrolled in different chemotherapy cycles, finally, there were a total of 190 cases. Among them, the olanzapine group had 94 cases and the aprepitant group had 96 cases. The baseline characteristics of the two groups are comparable, as shown in Table 1. This study has obtained the informed consent of the subjects and their relatives. Ethics approval for this study has been provided by Ordos Central Hospital and registration in China Clinical Trial Registration Center has been completed (Registration number:ChiCTR20000368269 (25/08/2020)). The study has followed CONSORT guidelines and the protocol was performed in accordance with the Declaration of Helsinki [18].

**Table 1:** Patients baseline characteristics (n(%))

Characteristics	olanzapine (n=94)	aprepitant (n=96)	P
Age(years)	59.44±9	59.65±9.968	0.406
≥55	72(76.60)	71(73.96)	0.674
Gender			0.643
Female	38(40.43)	42(43.75)	
Male	56(59.57)	54(56.25)	
History of motion sickness	16(17.02)	13(13.54)	0.505
History of female pregnancy vomiting	5(5.32)	10(10.41)	0.193
Alcohol use			0.598
No Consumption	50(53.19)	44(45.83)	
<4 drinks per week	27(28.72)	32(33.33)	
≥4drinks per week	17(18.09)	20(20.83)	
Smoking Index			0.508
No Smoking	35(37.23)	42(43.75)	
0~400	13(13.83)	9(9.38)	
≥400	46(48.94)	45(46.87)	
Type of malignance			0.735
Lung cancer	34(36.17)	37(38.54)	
Others	60(63.83)	59(61.46)	
Chemotherapy Cycle			0.378
First- Cycle	23(24.47)	31(32.29)	
Second - Cycle	28(29.79)	22(22.92)	
Third - Cycle	15(15.56)	20(20.83)	
≥Fouth- Cycle	28(29.78)	23(23.96)	

### Research methods

All patients in the group received a multi-day chemotherapy regimen of cisplatin, and the total dose of cisplatin was calculated according to 75 mg/m2, which was divided into days d1-3. The triple antiemetic regimen in the olanzapine group was: olanzapine 5 mg for 1–4 days, dexamethasone 10 mg for 1–3 days, and tropisetron 5 mg for 1–3 days. The triple antiemetic regimen of the aprepitant group was: aprepitant 125 mg on day 1 and 80 mg on day 2 and 3, dexamethasone 5 mg on day 1–3, and tropisetron 5 mg on day 1–3. Studies have shown that aprepitant can moderately inhibit CYP3A4 enzymes, interfere with the pharmacokinetics of dexamethasone and increase its blood concentration. So in contrast to the olanzapine group, the dexamethasone dose was halved in the aprepitant group [19]. (2) A total of 89 patients’ 5ml peripheral blood was collected on the day of chemotherapy and stored at -80 °C. BGI TECH SOLUTIONS (BEIJING LIUHE) CO.,Ltd. was entrusted to use MassARRAY SNP genotyping technique to sequence and type the following SNP sites: rs1062613, rs1176719, rs1176722, rs2276305, rs4938058, rs909411, rs1176713, rs1176744, rs12795805, rs2276303, rs3758987, rs45460698, rs7943062, rs11615, rs3212986, rs25487, rs744154, rs1801131, rs2231142, rs2238476, rs246240, rs2231137, rs2234978, rs2530797, rs3755468, rs3771836, rs17838409, rs2111375, rs3821313, rs6715729, rs16947, rs3892097, rs1065852, rs3918290, rs67376798, rs671, rs6766410, rs1801133, rs1801244, rs2854344, rs6443930, rs956304. Peripheral blood samples (5 mL) were collected from all subjects by a professional technician using a vacutainer and placed into tubes containing EDTA. We used a commercial DNA extraction kit (ZhongkeBio Medical Technology Co., Nanjing, China) to extract DNA from blood samples, according to the manufacturer’s protocol. DNA concentration and purity were evaluated using a NanoDrop2000 (ThermoFisher Scientific, Waltham, MA), and all samples met the quality requirements (OD 260/280 = 1.6–2.2). SNP detection primers were designed using Agena Bioscience Assay Designer4.0 software (https://agenacx.com/online-tools/) and synthesized by Thermo Fisher Scientific Inc. SNPs were genotyped using an Agena MassARRAY RS1000 (Agena, San Diego, CA, USA), according to the standard recommended instructions. Agena Bioscience 4.0 software was used to analyze and manage data.

### Evaluation indicators

After the start of chemotherapy, daily ward rounds are conducted or patient diaries are distributed to record nausea and vomiting within 0-120 h, their frequency, intensity, and adverse reactions. We also guide patients to fill in the FLIE scale. As evaluation indicators, TP was evaluated in three phases: acute (0-24 h), delayed (25-120 h) and overall (0-120 h). An individual with TP had no vomiting or severe retching that required rescue measures, with a maximum nausea score of ≤ 25 mm on the 100 mm Nausea Rating Scale.

### Statistical methods

The data were analyzed and processed using SPSS 25.0 statistical software. The baseline characteristics of the two groups were compared by mean ± standard deviation (± s), independent sample t-test and chi-square test. The χ2 test was used to analyze whether the genotype distribution conformed to the Hardy-Weinberg genetic balance law. The χ2 test was used for univariate analysis, Fisher’s exact test was used when the theoretical frequency was less than 5. Multivariate analysis was performed using binary logistic regression. P < 0.05 means the difference is statistically significant.

## Results

### Hardy-Weinberg balance test

In this study, except for *HTR3B* rs1062613 and Serotonin transporter Promoter rs956304 gene loci, the detection rates were 29% and 68%, respectively, and the detection rates of other SNP loci were ≥ 94%. The Chi-square test found that, except for the following 6 gene loci, the distribution frequencies of the other loci were consistent with the Hardy-Weinberg equilibrium law (*P* > 0.05). These 6 loci include: *TACR1* SNP (rs3821313), *CYP2D6* SNPs (rs3892097, rs1065852), *DPYD* SNPs (rs3918290, rs67376798), *RB1/LPAR6* SNP (rs2854344). The agreement of the Hardy-Weinberg equilibrium law suggests that the samples come from the same Mendelian group and are representative of the group.

### Single factor test of CINV association analysis

As shown in Table 2, the results of the χ2 test in the olanzapine group showed that increased TP in the acute phase was associated with the *HTR3A* rs1176719 non-GG genotype (*P* = 0.000) and rs2276303 GG (*P* = 0.016) genotype. Increased TP in the delayed phase was associated with *HTR3A* rs1176719 non-GG (*P* = 0.002), *ERCC1* rs3212986 CC (*P* = 0.018), *ERCC4* rs744154 non-CC (*P* = 0.003) genotypes. The χ2 test results of the aprepitant group showed that the increased TP in the acute phase was associated with the *MTHFR* rs1801131 TT(*P* = 0.0029) genotype, and the increased TP in the delayed phase was associated with *HTR3A* rs1062613 CC (*P* = 0.002), *HTR3B* -100-102AAG deletion wild type (*P* = 0.047), rs7943062 GG (*P* = 0.010), *HTR3C* rs6766410 non-CC (*P* = 0.013) and *MTHFR* rs1801131 TT (*P* = 0.003) genotypes.

**Table 2:** Univariate analysis of the relationship between TP and SNP alleles in olanzapine and aprepitant group at each stage

Polymorphism		Genotype	Olanzapine regimen(n)			Aprepitant regimen(n)		
			Acute Phase	Delayed Phase	Overall Phase	Acute Phase	Delayed Phase	Overall Phase
HTR3A	rs1062613	CC	15/15	9/15	9/15	19/19	14/19	14/19
		Non-CC	13/13	8/13	8/13	10/11	1/11	1/11
		*P*	1.000	1.000	1.000	0.367	0.002	0.002
	rs1176719	GG	61/63	36/63	36/63	63/69	37/69	37/69
		Non-GG	17/30	27/30	17/30	24/25	17/25	17/25
		*P*	0.000	0.002	0.002	0.748	0.245	0.245
	rs1176722	GG	75/79	46/79	46/79	79/85	50/85	50/85
		Non-GG	13/14	7/14	7/14	8/9	4/9	4/9
		*P*	0.566	0.771	0.771	0.518	0.635	0.635
	rs909411	GG	66/69	40/69	40/69	70/76	42/76	72/76
		Non-GG	22/24	13/24	13/24	17/18	12/18	12/18
		P	0.826	0.813	0.813	1.000	0.436	0.436
	rs1176713	AA	59/62	36/62	36/62	58/63	35/63	35/63
		Non-AA	29/32	17/32	17/32	31/33	20/33	20/33
		P	0.684	0.667	0.667	1.000	0.670	0.670
	rs2276303	GG	73/75	43/75	43/75	75/82	44/82	44/82
		Non-GG	15/19	10/19	10/19	14/14	11/14	11/14
		P	0.016	0.798	0.798	0.562	0.142	0.142
HTR3B	rs2276305	GG	62/66	37/66	37/66	51/56	28/56	28/56
		Non-GG	26/27	16/27	16/27	36/38	26/38	26/38
		P	1.000	0.821	0.821	0.792	0.092	0.092
	rs4938058	AA	53/55	32/55	32/55	49/53	31/53	31/53
		Non-AA	35/38	21/38	21/38	35/37	21/37	21/37
		P	0.669	0.833	0.833	1.000	1.000	1.000
	rs1176744	AA	50/53	29/53	29/53	49/53	30/53	30/53
		Non-AA	36/39	22/39	22/39	39/42	24/42	24/42
		P	1.000	1.000	1.000	1.000	1.000	1.000
	rs12795805	TT	51/54	29/54	29/54	51/55	31/55	31/55
		Non-TT	37/40	24/40	24/40	38/41	24/41	24/41
		P	1.000	0.674	0.674	1.000	0.838	0.838
	rs3758987	TT	48/51	29/51	29/51	51/53	32/53	32/53
		Non-TT	40/43	24/43	24/43	38/43	23/43	23/43
		P	1.000	0.180	0.180	0.281	0.538	0.538
		Variants (del/del+del/ins)	19/19	10/19	10/19	29/31	13/31	13/31
		Wild type (ins/ins)	68/73	43/73	43/73	60/65	42/65	42/65
		P	0.545	0.795	0.795	1.000	0.047	0.047
	rs7943062	GG	65/69	42/69	42/69	71/76	49/76	49/76
		Non-GG	23/25	11/25	11/25	18/20	6/20	6/20
		P	1.000	0.164	0.164	0.968	0.010	0.010
HTR3C	rs6766410	CC	13/14	6/14	6/14	9/11	2/11	2/11
		Non-CC	75/79	47/79	47/79	78/83	52/83	52/83
		P	0.566	0.380	0.380	0.189	0.013	0.013
HTR3D	rs6443930	CC	22/25	13/25	13/25	27/29	20/29	20/29
		Non-CC	66/69	40/69	40/69	61/66	35/66	35/66
		P	0.388	0.644	0.644	1.000	0.179	0.179
Serotonin transporter Promoter	rs956304	TT	63/65	44/65	44/65	64/69	42/69	42/69
		Non-TT	1/2	0/2	0/2	4/4	1/4	1/4
		P	0.088	0.114	0.114	1.000	0.371	0.371
ERCC1	rs11615	GG	70/74	42/74	42/74	55/59	33/59	33/59
		Non-GG	18/19	11/19	11/19	32/35	21/35	21/35
		P	1.000	1.000	1.000	1.000	1.000	1.000
	rs3212986	CC	37/37	27/37	27/37	50/52	32/52	32/52
		Non-CC	51/56	26/56	26/56	37/42	22/42	22/42
		P	0.162	0.018	0.018	0.278	0.407	0.407
ERCC4	rs25487	CC	51/53	27/53	27/53	51/54	29/54	29/54
		Non-CC	37/40	26/40	26/40	36/40	25/40	25/40
		P	0.746	0.208	0.208	0.679	0.409	0.409
	rs744154	CC	11/12	2/12	2/12	1/1	1/1	1/1
		Non-CC	76/80	51/80	51/80	88/95	54/95	54/95
		P	0.511	0.003	0.003	1.000	1.000	1.000
MTHFR	rs1801131	TT	60/63	37/63	37/63	63/65	43/65	43/65
		Non-TT	27/29	15/29	15/29	21/26	8/26	8/26
		P	1.000	0.651	0.651	0.029	0.003	0.003
	rs1801133	AA	28/29	17/29	17/29	23/23	16/23	16/23
		Non-AA	60/65	36/65	36/65	66/73	39/73	39/73
		P	0.748	0.825	0.825	0.279	0.229	0.229
ABCG2	rs2231142	GG	47/50	28/50	28/50	44/47	26/47	26/47
		Non-GG	41/43	25/43	25/43	43/47	28/47	28/47
		P	1.000	1.000	1.000	1.000	0.327	0.327
	rs2238476	GG	76/80	45/80	45/80	78/85	49/85	49/85
		Non-GG	12/13	8/13	8/13	9/9	5/9	5/9
		P	0.374	0.772	0.772	1.000	1.000	1.000
	rs246240	AA	28/28	15/28	15/28	29/32	18/32	18/32
		Non-AA	60/65	38/65	38/65	58/62	36/62	36/62
		P	0.314	0.820	0.820	0.923	1.000	1.000
	rs2231137	CC	35/37	21/37	21/37	44/49	28/49	28/49
		Non-CC	53/57	32/57	32/57	45/47	27/47	27/47
		P	1.000	1.000	1.000	0.467	1.000	1.000
FAS / CD95	rs2234978	CC	81/85	49/85	49/85	75/81	49/81	49/81
		Non-CC	7/8	4/8	4/8	12/13	5/13	5/13
		P	0.369	0.965	0.965	1.000	0.226	0.226
CCL2	rs2530797	TT	48/50	28/50	28/50	40/43	26/43	26/43
		Non-TT	40/43	25/43	25/43	47/51	28/51	28/51
		P	0.862	1.000	1.000	1.000	0.677	0.677
TACR1	rs3755468	CC	23/25	15/25	15/25	9/9	5/9	5/9
		Non-CC	61/63	36/63	36/63	75/82	46/82	46/82
		P	0.680	0.155	0.155	1.000	1.000	1.000
	rs3771836	TT	45/45	30/45	30/45	50/54	32/54	32/54
		Non-TT	43/48	23/48	23/48	37/40	22/40	22/40
		P	0.077	0.094	0.094	1.000	0.833	0.833
	rs17838409	CC	84/89	53/89	53/89	89/95	55/95	55/95
		Non-CC	4/5	0/5	0/5	0/1	0/1	0/1
		P	0.286	0.014	0.014	0.294	0.427	0.427
	rs2111375	GG	46/48	26/48	26/48	53/58	31/58	31/58
		Non-GG	42/46	27/46	27/46	36/38	24/38	24/38
		P	0.634	0.683	0.683	0.828	0.402	0.402
	rs3821313	GG	60/62	39/62	39/62	66/73	41/73	41/73
		Non-GG	20/24	8/24	8/24	20/20	11/20	11/20
		P	0.085	0.017	0.017	0.336	1.000	1.000
	rs6715729	AA	24/27	16/27	16/27	21/23	12/23	12/23
		Non-AA	64/67	37/67	37/67	68/73	43/73	43/73
		P	0.469	0.820	0.820	1.000	0.633	0.633
CYP2D6	rs16947	GG	54/57	36/57	36/57	58/60	35/60	35/60
		Non-GG	34/37	17/37	17/37	31/36	20/36	20/36
		P	0.905	0.136	0.136	0.128	0.833	0.833
ALDH2	rs671	GG	66/70	39/70	39/70	61/65	39/65	39/65
		Non-GG	21/22	14/22	14/22	24/26	15/26	15/26
		P	1.000	0.623	0.623	1.000	1.000	1.000
ATP7B	rs1801244	CC	29/31	19/31	19/31	36/37	24/37	24/37
		Non-CC	59/63	34/63	34/63	53/59	31/59	31/59
		P	1	0.517	0.517	0.334	0.291	0.291

### Multivariate logistic regression analysis of CINV association analysis

In order to adjust possible confounding factors, the genotypes with statistically significant differences in single factor analysis of grouping, gender, acute phase and delayed phase were further included in the multivariate Logistic regression model for multivariate analysis. Table 3 shows that *HTR3B* rs7943062 GG genotype, *HTR3C* rs6766410 non-CC genotype and *MTHFR* rs1801131 TT genotype are potential independent protective factors for the TP rate of CINV in the delayed phase.

**Table 3:** Multivariate logistic regression for TP and some SNPs during acute and delayed phases

Clinical factors		Acute Phase			Delayed Phase		
		OR	95% CI	P value	OR	95% CI	*P* value
Group		0.649	0.189-2.231	0.493	0.14	0.015-1.337	0.088
Gender		0.661	0.191-2.292	0.514	0.123	0.0040-4.002	0.238
HTR3A	rs1062613 CC vs. Non-CC				1.06	0.019-58.503	0.977
	rs1176719 GG vs. Non-GG	1.355	0.156-11.800	0.783	0.076	0.001-9.345	0.293
	rs2276303 GG vs. Non-GG	0.294	0.027-3.193	0.315			
HTR3B	rs45460698 -100_-102AAG deletion variants vs. wild type				1.297	0.092-18.193	0.847
	rs7943062 GG vs. Non-GG				0.004	0.000-0.221	0.007
HTR3C	rs6766410 CC vs. Non-CC				41645.423	19.065-90971913.963	0.007
ERCC1	rs3212986 CC vs. Non-CC				22.888	0.575-910.550	0.096
ERCC4	rs744154 CC vs. Non-CC				19,150,000,000	0.000-.	1
MTHFR	rs1801131 TT vs. Non-TT	0.263	0.074-0.926	0.038	0.005	0.000-0.807	0.041

## Discussion

This study used a prospective randomized controlled trial to observe the antiemetic effect of chemotherapy patients with different genetic polymorphisms, and most similar studies at home and abroad have chosen this research method. In this study, we explored the association between the TP rate of olanzapine or aprepitant triple antiemetic regimen to prevent multi-day cisplatin-induced CINV and 36 CINV-related gene polymorphisms. The results showed that multiple polymorphisms were associated with CINV. The results of univariate analysis showed that CINV was correlated with *HTR3A* SNPs (rs1176719, rs2276303, rs1062613), *HTR3B* SNPs (rs45460698, rs7943062), *HTR3C* SNPs (rs6766410), *ERCC1* SNPs (rs3212986), *ERCC4* SNPs (rs744154) and *MTHFR* SNPs (rs1801131). After multivariate correction analysis, excluding the influence of olanzapine and aprepitant grouping and gender, it was shown that *HTR3B* rs7943062, *HTR3C* rs6766410, and *MTHFR* rs1801131 gene polymorphisms were associated with CINV. Patients with *HTR3B* rs7943062 GG genotype had a lower risk of delayed CINV after chemotherapy than patients with non-GG genotype. Patients with *HTR3C* rs6766410 CC genotype had a higher risk of delayed CINV after chemotherapy than patients with non-CC genotype. Patients with *MTHFR* rs1801131 TT genotype had a lower risk of delayed CINV after chemotherapy than patients with non-TT genotype. Therefore, the above SNPs may serve as genetic markers of CINV association and become potential genetic targets for CINV prevention and treatment.

rs1062613 is located in the promoter region of the *HTR3A* receptor and can regulate the expression of the entire receptor gene [20]. The study of Kaiser et al. did not find any correlation between *HTR3A* rs1062613 and CINV [21]. Another pharmacogenetic study of nausea and vomiting in pregnancy found that patients carrying the rs1062613 non-CC variant allele had poorer nausea and vomiting scores. This is consistent with our univariate analysis results that rs1062613 non-CC had a lower TP in the delayed phase [22]. A foreign basic research showed that the C allele of rs1062613 was related to the low expression of serotonin, which also supported our findings [23].

The results of univariate analysis in this study showed that the non-GG genotype of *HTR3A* rs1176719 in the olanzapine group was related to the TP rate in the overall phase, and the rs2276303 GG genotype was related to the TP rate in the acute phase. However, the study by Kaiser et al. did not find the correlation between rs1176719 and rs2276303 SNPs and CINV [21]. Both rs1176719 and rs2276303 are located in the intron region of the *HTR3A* receptor. Although they are not directly involved in protein translation, introns, as the main components of broken genes, may play an important role in gene expression. Studies have found that intron mutations can produce multiple different proteins from the same gene due to different splicing sites after transcription. Abnormal expression of protein may activate some recessive splice sites, leading to disease [23, 24]. However, so far, no other studies on the relationship between the above gene loci and CINV have been retrieved, and the above results in this study need to be further verified in other races and with a larger sample size.

The − 100_-102AAG deletion (rs45460698) located in the promoter region is a common polymorphism in the 5-HT3B subunit. Tremblay et al. found that in all Caucasian patients who experienced CINV, the frequency of vomiting was significantly increased in patients with − 100_-120delAAG deletion [25]. Another Korean study also found that *HTR3B* -100_-102delAAG deletion variants had higher acute nausea and vomiting than wild-type patients [26]. These studies are consistent with our univariate analysis results, the wild-type gene has a higher TP rate, and the *HTR3B* -100_-102delAAG genotype may be an independent factor affecting CINV. For patients carrying the − 100_-102delAAG deletion mutation, it may be considered to add or alternately use antiemetic drugs on the basis of 5-HT3 antagonists to control acute vomiting, but it is still necessary to expand the sample size and further determine the population.

SNP rs7943062 is located at the mutation site in the 3’ non-coding region of the *HTR3B* gene. They are not directly involved in the translation process of the protein, and may change the expression and activity of the 5-HT3B receptor by affecting the translation regulation process. We found that the TP rate of CINV in the delayed phase of patients with GG genotype of this gene locus was higher than that of patients with non-GG genotype. In a study by Perwitasari et al. on 202 Indonesian patients using cisplatin as monotherapy or in combination with chemotherapy, 8 mg ondansetron and 8 mg dexamethasone were routinely given intravenously as CINV prophylaxis before chemotherapy. However, the results did not show that the rs7943062 gene polymorphism was associated with CINV [17]. We analyzed that there may be the following reasons: Perwitasari et al. did not control age, gender and other non-research factors that may affect CINV, ethnic differences and different prevention programs will affect the research results [27].

The non-synonymous SNP of *HTR3C* rs6766410 results in the replacement of aspartic acid at position 163 with lysine. This may affect the electrostatic potential at the interface between two adjacent subunits of the serotonin receptor, thereby indirectly changing the structure of the receptor [28]. In patients with primary breast cancer treated with epirubicin (with or without cyclophosphamide)-naive chemotherapy, Fasching et al. showed that homozygosity for the rare C allele at rs6766410 was associated with emesis in the acute phase. This supports the findings of this study that the *HTR3C* rs6766410 non-CC genotype has a higher TP rate in the delayed phase [29]. The study by Mukoyama et al. also showed consistent results [16]. In contrast, homozygosity for the CC allele was found to be associated with reduced severity of CINV in the acute phase in the study by Pud et al. [30]. The study by Ward et al. did not show a correlation between the two [31]. Three studies involving the same SNP showed three completely different results. It can be seen that the reasons for the induction of CINV are complex, not only involving the regulation of the nervous system, but also affected by various external environmental factors. In addition, the intrinsic association between clinical outcomes and gene expression is influenced by statistical methods and the sample size of the study population. Therefore, a larger, well-designed prospective randomized controlled study is needed to further clarify its relationship.

A study by Yokoi et al. examined 156 Japanese patients receiving cisplatin chemotherapy. In multivariate logistic regression analysis, *ERCC1* rs3212986 AA genotype was significantly associated with acute phase CINV. This is consistent with the findings of this study that rs3212986 CC type has a higher delay phase TP rate [12]. The reason may be that the *ERCC1* rs3212986 A allele can reduce the expression of its encoded DNA endonuclease in normal gastrointestinal tissues, which can promote the dysfunction of small intestinal cells caused by anticancer drugs, thereby inducing CINV [12].

Our univariate analysis found that olanzapine-treated patients had a delayed TP rate associated with the *ERCC4* rs744154 non-CC genotype. However, a study of gene polymorphisms and chemotherapy toxicity in patients with non-small cell lung cancer treated with platinum and paclitaxel chemotherapy showed that there was no correlation between rs744154 SNP and CINV [32]. Since *ERCC4* is involved in the metabolism of platinum, genetic variation of this gene may affect the pharmacokinetic and pharmacodynamic pathways of platinum, further leading to differences in response and tolerance among patients [33]. However, the distribution of variant genes in different populations may lead to differences in chemotherapy toxicity in Asians and Caucasians.

This study found that the incidence of CINV in patients with TT genotype at *MTHFR* rs1801131 in the aprepitant group was lower than that in patients with non-TT genotype, which was consistent with the results reported by Gao et al. in Chinese gastric cancer patients [11]. It is suggested that the T allele of rs1801131 at this locus may be a protective factor for CINV in Chinese population. No relevant foreign literature has been retrieved yet, suggesting that this locus has value for research in different populations.

This study has not found any correlation between the following genes and CINV: *HTR3A* SNPs (rs1176722, rs909411, rs1176713), *HTR3B* SNPs (rs2276305, rs4938058, rs1176744, rs12795805, rs3758987), *HTR3D* SNPs (rs6443930), Serotonin transporter Promoter SNPs (rs956304), *ERCC1* SNPs (rs11615), *ERCC4* SNPs (rs25487), *MTHFR* SNPs (rs1801133), *ABCG2* SNPs (rs2231142, rs2238476, rs246240, rs2231137), *FAS/CD95* SNPs (rs2234978), *CCL2* SNPs (rs2530797), *TACR1* SNPs (rs3755468, rs3771836, rs17838409, rs2111375, rs3821313, rs6715729), *CYP2D6* SNPs (rs16947, rs3892097, rs1065852), *DPYD* SNPs (rs3918290, rs67376798), *ALDH2* SNPs (rs671), *ATP7B* SNPs (rs1801244), *RB1/LPAR6* SNPs (rs2854344). Although some domestic and foreign studies have found that some of these genes are associated with CINV, there is a lack of confirmation from strictly designed clinical research data. Further research on the functional characteristics of these SNPs is needed to verify their association with CINV.

The results of this experiment can conclude that the gene polymorphisms of *HTR3A*, *HTR3B*, *HTR3C*, *ERCC1*, *ERCC4* and *MTHFR* may be involved in the occurrence and development of CINV. This is basically consistent with the previous research results at home and abroad, but when it comes to the relationship between some genotypes and clinical symptoms, the research results are not completely the same in different populations. The prevention and treatment of CINV is affected by multiple factors such as race, emetogenic drugs, prevention programs, and evaluation indicators. Therefore, the interpretation and promotion of clinical research results need to be cautious.

This study has some limitations. First, this study did not measure antiemetic drug concentrations in plasma or cerebrospinal fluid, and no pharmacokinetic information was available. Therefore, we were unable to elucidate the basis of SNP action and the mechanism of response to antiemetics Secondly, the sample size used to analyze gene polymorphisms and TP rates is relatively small, so clinical studies with larger sample sizes are needed to provide stronger evidence support in the future.

## Conclusion

In summary, we examined the correlation between related gene polymorphisms and the TP rate of CINV. We also confirmed the relationship between the TP rate and each gene locus in patients in northern China who received multi-day chemotherapy with cisplatin. This study reveals that *HTR3B* rs7943062 GG genotype, *HTR3C* rs6766410 non-CC genotype, and *MTHFR* rs1801131 TT genotype are independent protective factors for delayed CINV in northern Chinese population. Our research, by identifying the risk of CINV in patients with different gene polymorphisms on the basis of pharmacogenetics, has certain value for the screening of CINV susceptible population in China, which is helpful to prevent CINV and improve the quality of life of cancer patients. In the future, the development of simple kits can help to quickly screen CINV high-risk groups to guide the use of chemotherapy regimens or antiemetic regimens.

### Electronic supplementary material

Below is the link to the electronic supplementary material.

Supplementary Material 1

## Data Availability

The datasets supporting the conclusions of this article are included within the article.
