# Supplementary material for: A randomized trial evaluating the association between related gene polymorphism and nausea and vomiting induced by cisplatin multi-day chemotherapy
Source: BMC Med Genomics. 2023 Nov 3;16:276. doi: 10.1186/s12920-023-01719-0 (PMC10625179; doi:10.1186/s12920-023-01719-0)

**Supplementary material**

Because this study entrusts the gene testing company to carry out genetic analysis, and is limited to space and research focus, we didn’t involve more detailed experimental content in this paper. I hope to get your understanding. Here are the overview of Massarray SNP typing Technology flow(Figure 1: Massarray SNP typing Technology flow). But in the future, we will research on this topic and hope you may understand our difficulty at this stage.

Figure 1: Massarray SNP typing Technology flow


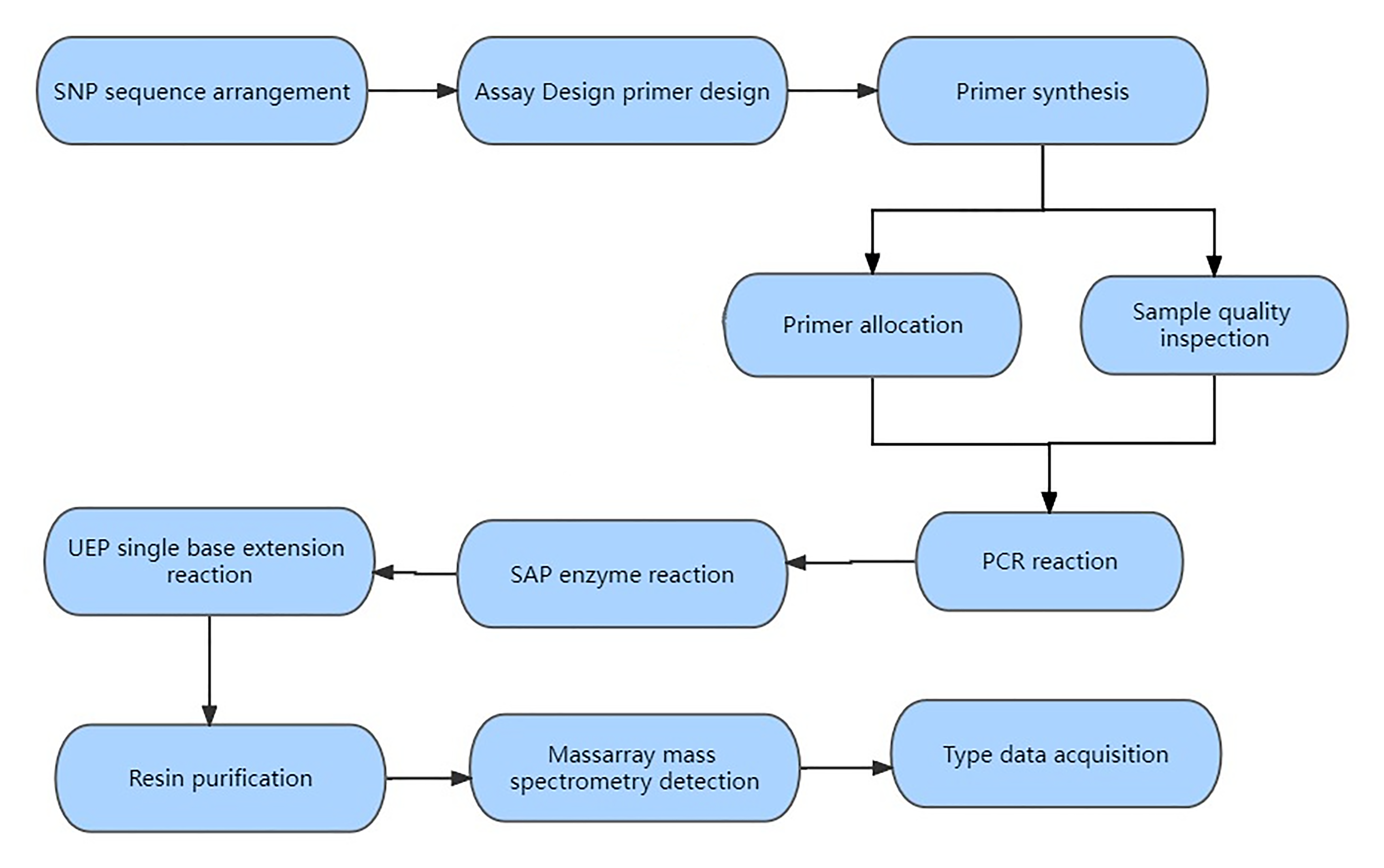

Supplement: Supplementary file 1 — Supplementary Material 1 [file 12920_2023_1719_MOESM1_ESM.docx]
